# Diode‐based transmission detector for IMRT delivery monitoring: a validation study

**DOI:** 10.1120/jacmp.v17i5.6204

**Published:** 2016-09-08

**Authors:** Taoran Li, Q. Jackie Wu, Thomas Matzen, Fang‐Fang Yin, Jennifer C. O'Daniel

**Affiliations:** ^1^ Department of Radiation Oncology Duke University Medical Center Durham NC USA; ^2^ ScandiDos AB Uppsala Sweden

**Keywords:** transmission detector, real‐time quality assurance, IMRT quality assurance, *in vivo* dosimetry

## Abstract

The purpose of this work was to evaluate the potential of a new transmission detector for real‐time quality assurance of dynamic‐MLC‐based radiotherapy. The accuracy of detecting dose variation and static/dynamic MLC position deviations was measured, as well as the impact of the device on the radiation field (surface dose, transmission). Measured dose variations agreed with the known variations within 0.3%. The measurement of static and dynamic MLC position deviations matched the known deviations with high accuracy (0.7–1.2 mm). The absorption of the device was minimal (∼ 1%). The increased surface dose was small (1%–9%) but, when added to existing collimator scatter effects could become significant at large field sizes ($\geq 30\times 30\;{\text{cm}}^{2}$). Overall the accuracy and speed of the device show good potential for real‐time quality assurance.

PACS number(s): 87.55.Qr

## I. INTRODUCTION

Complicated and hypofractionated treatment procedures, such as stereotactic body radiation therapy (SBRT), have recently gained popularity in the field of radiation oncology.^(1)^ Larger fractional dose and smaller total treatment fractions require that the daily dose be delivered accurately with very little room for errors. Advanced technologies such as online adaptive radiation therapy also demand real‐time monitoring and verification of the delivery process, including machine output and MLC position/movement.^2^, ^3^ In addition to traditional pretreatment QA, being able to verify whether the radiotherapy plan is accurately delivered by monitoring the machine output, dose rate, and MLC positions while the delivery is occurring has become the key to ensure high‐quality care for the patient receiving tight‐margin, intensity‐modulated radiation therapy(IMRT) and SBRT. Therefore, the radiation therapy field is in great need for real‐time treatment delivery monitoring devices and technologies.

Currently, there are three main categories of real‐time monitoring solutions: linear accelerator monitoring, transit detectors, and transmission detectors. In the first group, an independent software program monitors the linear accelerator parameters as the treatment is being delivered.^4^, ^5^ This relies on the linear accelerator to accurately report its parameters. In the second group, a 2D transit detector is placed so that the radiation will first penetrate the patient, then the detector.^6^, ^7^ Typically an electronic portal imager (EPID) is used as the detector. A separate software program then interprets the EPID data to estimate the dose delivered to the patient. The presence of the moveable patient body in the detection path complicates the analysis, but is beneficial in that the position of the patient may be taken into account when determining the delivered dose. In the third group, a thin transmission detector (TRD) is inserted between the radiation source and the patient. This allows for independent monitoring of the radiation delivery, including MLC leaf motion. The majority of current TRDs utilize ionization chambers in different forms, including wires,^8^, ^9^, ^10^ plane parallel chambers,^11^, ^12^, ^13^ and a large area wedge ion chamber.^14^, ^15^ One optical‐attenuation‐based TRD using scintillating fibers is also described.^(16)^ The ability of these TRDs to detect small positional changes across the two‐dimensional treatment fields is limited by the detector's shape (one‐dimensional wires/fibers) and resolution. Another previously published TRD utilized diodes with a wide 1 cm spacing, but only a general analysis of the performance of the diodes in a radiation environment was presented.^(17)^ In order to adequately sample a realistic photon dose profile, including for IMRT and VMAT dose deliveries, a minimum sampling step width of 2.5 mm is required per the Nyquist theorem.^18^, ^19^ In this study, we examine a new TRD design utilizing high‐resolution (2.5 mm) diodes for patient‐specific, real‐time quality assurance of IMRT delivery. The purpose of this study is to comprehensively evaluate this novel 2D TRD in the context of treatment delivery monitoring. Characteristics evaluated include the TRD's impact on the radiation treatment, detection accuracy for machine output deviations, and detection accuracy and precision for static and dynamic MLC position errors.

## II. METHODS

### A. Diode‐based transmission detector

**Figure 1:**
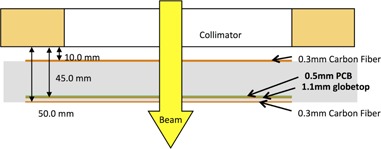
Cross‐sectional diagram of the diode‐based transmission detector.

The 2D TRD array consists of 4,040 p‐type diode detectors, each with an active area of 1 mm diameter (Delta^4^ AT, also referred to as Delta^4^ Discover, ScandiDos AB, Uppsala, Sweden). This diode array covers a dimension of 25 cm by 20 cm when projected to the isocenter plane at 100 cm source‐to‐axis distance (SAD). Along the MLC leaf travel direction (X direction), the diodes are spaced 1.6 mm apart (2.5 mm apart projected to the isocenter plane); perpendicular to the leaf travel direction (Y direction), diodes are spaced at 3.2 mm intervals (5.0 mm intervals projected to the isocenter plane). For MLCs of 5 mm width at isocenter, this arrangement allows each leaf‐pair in the active detection area to be monitored with one unique line of diode arrays, at a 2.5 mm sampling spacing. The overall detector thickness is 4.0 cm (Fig. 1) with a source‐to‐device distance of 63.6 cm for Varian linear accelerators. The wireless TRD is battery‐driven with an operational capacity of approximately about 4 hrs (four batteries of 1 hr capacity each; may be removed individually for recharging). Communication is via Wi‐Fi to the QA software on a customer computer. For a light field check the TRD can be slid out while remaining fixed to the gantry. The linear accelerator's optical distance indicator is visible both in the default and extended positions. Although heavy for a radiation accessory (10 kg without batteries plus 0.5 kg per battery), it can be mounted/unmounted in less than 1 min to/from the gantry.

**Figure 2:**
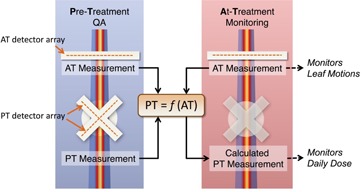
Overview of the TRD process including both pretreatment (PT) QA and at‐treatment (AT) monitoring.

The novel TRD is an add‐on component to an existing 3D QA device (Delta^4^ PT, ScandiDos) described by Sadagopan et al.^(20)^ It relies on the QA phantom for a pretreatment baseline measurement and shares the same software to perform dosimetric evaluations during treatment. During the pretreatment QA, the QA software takes input from both the TRD and 3D QA device to generate an internal model that links these two measurements. During the actual treatment delivery, only the TRD is mounted on the gantry. The measurement from the TRD device is sent at 25 ms intervals to the QA software via Wi‐Fi connection. The model built during the pretreatment QA phase is then used to calculate a virtual dose measurement based on TRD input (Fig. 2). This virtual measurement can be analyzed using the same software as the pretreatment IMRT QA, comparing calculated to measured 3D dose distributions. In addition to composite 3D dose verification, the measurement from the TRD can also directly be used to verify dynamic leaf motion based on its real‐time 2D signal.

### B. Impact on beam quality

The TRD is placed in the beam path between the source and the patient, and therefore can potentially impact the beam quality that the patient receives. To quantify this impact, beam transmission factors and relative surface doses were measured with the TRD mounted in the path of 6 MV and 15 MV photon beams on a Varian 2100iX linear accelerator with Millennium MLC (Varian Medical Systems, Palo Alto, CA).

A motorized water phantom (Blue Phantom, IBA Dosimetry, Bartlett, TN) with a CC13 ion chamber (IBA Dosimetry) was placed in the beam at a distance of SSD=90 cm. Percentage depth‐dose scans were performed for 6 MV and 15 MV photon beams with field sizes of 4×4,10×10, and $30\times 30\;{\text{cm}}^{2}$. In‐plane and cross‐plane profiles for both energies at depths of dmax and 10 cm for a field size of $30\times 30\;{\text{cm}}^{2}$ were also collected. A 2 mm diode detector (PFD^3G^, IBA Dosimetry) with an effective point of measurement of approximately 0.6 mm below its front surface was positioned at the water surface and dmax for surface dose measurements (normalized to dmax) and at 10 cm depth for transmission measurements (normalized to local dose).^(21)^ Measurements were done for field sizes (defined at 100 cm SSD) of 3×3,5×5,10×10,20×20,30×30, and $40\times 40\;{\text{cm}}^{2}$. The PFD^3G^ signal/100 MU for a given field size was collected with a Fluke electrometer model 35040 (Fluke Electronics Corporation, Everett, WA). Measurements with and without the TRD detector attached to the gantry were compared with each other.

### C. Error Simulation

Introduced error measurements were done on a pair of Varian 2100iX linear accelerators, with millennium MLC, at gantry 0° (IEC 621217), with 6 MV photons. First, the ability of the TRD to detect fluctuations in machine output was verified. Using a static MLC field, machine output errors were introduced by modifying the delivered MU to 1%–5% higher than planned (1‐5 MU increase from a reference 100 MU field). This set of MU error plans were used to assess the TRD's sensitivity to detecting and quantifying small output deviations between planning and delivery by comparing the TRD‐measured fluence during error‐bearing delivery vs. the baseline delivery.

**Figure 3:**
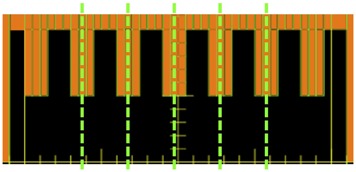
Illustration of the analyses for static MLC deviation and machine output deviation. Green dashed line indicates the 1D intensity profile used to assess the TRD's response to MLC shifts. All open areas (black) were used to measure machine output deviations simulated by increasing MUs at delivery.

Second, the TRD's ability to detect static MLC errors was evaluated using a comb‐shaped static MLC field, created by extending half of the MLC leaves half‐way into the radiation field (Fig. 3). MLC leaf position errors of 1–5 mm were introduced manually, retracting the extended leaves away from the central axis. The same error was applied to all MLC leaves for each measurement. The measured fluence of the error‐bearing delivery was compared to the measured fluence of the baseline (no shift) delivery by looking at a one‐dimensional profile along the direction of leaf movement (X direction).

**Figure 4:**
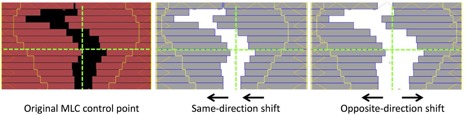
Illustration of dynamic MLC errors. Leaves are shifted in both same direction and opposite directions to simulate two different leaf‐motion deviation scenarios.

Third, dynamic IMRT delivery was used to test the TRD's response to manually introduced errors. In this case a single field from a prostate IMRT treatment plan was used. To simulate MLC motion errors, each control point in the original plan was shifted by 0.5 mm, 1 mm, 2 mm, 3 mm, and 5 mm in both the extension and retraction directions. If opposing leaves in the same leaf pair were shifted in the same direction relative to the middle position, the leaf opening was displaced towards one side (Fig. 4). If opposing leaves were both retracted by a certain amount, the leaf opening became larger than the planned size (Fig. 4). Reducing leaf openings was not tested due to the potential for leaf collisions. In this analysis, the raw TRD measurement was used to analyze for MLC motion deviations during dynamic delivery.

## III. RESULTS

### A. Impact on beam quality

Dose profiles (percentage depth dose, in‐plane, and cross‐plane) were virtually indistinguishable between measurements with and without the TRD in the beam path. The largest changes overall were a 0.5% decrease in the dose at 10 cm depth (median 0.0%), a 1.2 mm shift in the depth of maximum dose towards the surface (median 0.2 mm), a 0.3% decrease in flatness at 10 cm depth (median 0.0%), and a 0.5% increase in symmetry at a depth of dmax (median 0.05%).

**Figure 5:**
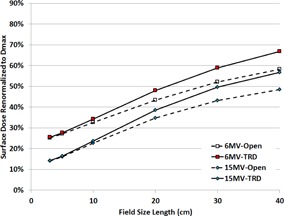
Surface dose with and without the presence of the TRD detector array.

The average transmission factor, due to the presence of the 2D transmission detector array, was 0.989±0.0015 for 6 MV and 0.993±0.0006 for 15 MV (one standard deviation (SD)). Figure 5 shows the surface dose increase for a range of beam energies and field sizes. The data points correspond to depth in water of approximately 0.6 mm. For field sizes less than $10\times 10\;{\text{cm}}^{2}$, the increase attributable to the detector is less than 1%. For the $40\times 40\;{\text{cm}}^{2}$ field size, an additional 8% (6 MV) and 9% (15 MV) surface dose was attributed to the addition of the TRD detector array. Table 1 provides a comparison of the transmission/surface dose increases due to this TRD (Delta^4^) to other published TRDs.

**Table 1:** Comparison of TRDs for 6 MV photons.

	*DAVID* ^a^	*Compass*	*IQM*	*Optical*	*Magic Plate*	*Delta* ^4^
Surface dose increase (10x10 cm^2^)	1.01	1.030	1.03	1.11	n/a^b^	1.017
Surface dose increase ($20\times 20\;{\text{cm}}^{2}$)	1.06	1.081	n/a^b^	1.21	1.034	1.046
Transmission factor	0.928–0.939	0.967±0.002	0.930	0.983±0.003	0.990±0.002	0.989±0.0015
TRD thickness	1.4 cm	11.0 cm	4.0 cm	0.32 cm	1.5 cm	5.0 cm
Device‐to‐surface distance	26 cm	25 cm	25.9 cm	26.2 cm	30.5 cm	26.4 cm
Measurement depth	1.5 mm	0.153 mm	n/a	0.01 mm	0.5 mm	0.6 mm
Depth of normalization	10 cm	dmax	dmax	10 cm	dmax	dmax
Reference	Poppe^(10)^	Venkataraman^(11)^	Islam^(14)^	Goulet^(16)^	Wong^(17)^	Current article

^a^ DAVID measurements at 70 cm SSD to maintain similar measurement depth; all others at 90 cm SSD. b n/a = not available in the published literature.

### B. Error Simulation

#### B.1 Output deviation

**Figure 6:**
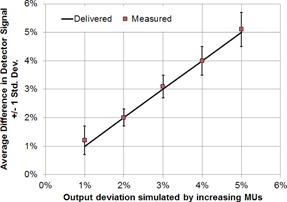
Comparison of measured and delivered beam output with 1%‐5% manually introduced deviation. Error bars were the standard deviation of multiple measurement points.

Figure 6 shows the detector's response to linear accelerator output deviation simulated by manually delivering 1%‐5% more MU. The measured signal at the detector was then compared to the original measurement. The MU linearity (measured during annual QA) was within 0.1% of the nominal MU, therefore the increase in MU can accurately simulate the machine output drift. The detector's average response agreed with the actual output increase within 0.3%.

#### B.2 Static MLC position deviation

**Figure 7:**
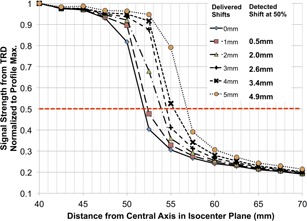
1D profile of TRD measurement along one leaf. Different lines represent multiple manually introduced static MLC leaf position deviations from 1 mm to 5 mm. The legend provides a comparison between delivered shift and detected shift based on the location of 50% signal strength.

Static MLC positioning errors were simulated by delivering plans with MLC leaves shifted from their original positions by 1 mm to 5 mm. To identify the leaf positions from the detector data, profiles along the direction of leaf motion were extracted from the 2D dose map at the center of each MLC moving leaf grouping (1.5 cm width at isocenter=3 MLC leaves) and compared between the plans. Due to the presence of MLC penumbra, the position of 50% signal intensity (as determined through linear interpolation from the measured data) was selected to represent the measured leaf end position. Figure 7 shows the six profiles corresponding to the original delivery (0 mm) and five deliveries with introduced 1–5 mm offsets. Each profile corresponds to an average of five lines of response in the TRD detector array (one per in‐field MLC group, every 3 cm). The six profiles are distinct; the measured leaf end positions are within 0.36 mm on average of the known offset (0.68 mm maximum discrepancy). Table 2 summarizes the average, maximum, and minimum detected errors for the five introduced MLC deviation levels from 1 mm to 5 mm. The small standard deviation indicates a measurement precision of at least 0.10 mm (one sigma) at different MLC locations.

**Table 2:** Average, maximum, minimum, and standard deviation (SD) of the static MLC deviation detection across five different leaf pairs as shown in Fig 7.

*Introduced Deviation (mm)*	*Measurement*			
	*Avg. (mm)*	*Min. (mm)*	*Max. (mm)*	*SD (mm)*
1.00	0.45	0.37	0.50	0.05
2.00	1.83	1.79	1.88	0.04
3.00	2.60	2.54	2.68	0.06
4.00	3.42	3.32	3.55	0.10
5.00	4.91	4.78	5.03	0.09

#### B.3 Dynamic MLC position deviation

**Figure 8:**
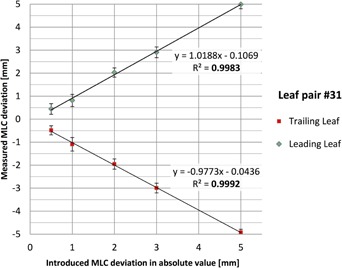
Detected vs. introduced MLC position errors during a dynamic IMRT delivery. Results for a representative leaf pair #31 near the center of the field are shown. Error bars show the standard deviation across all measurement time points during the beam delivery.

Figure 8 compares the detected MLC position deviations vs. the known deviations manually introduced in the dynamic IMRT treatment plan for one representative leaf pair near the center of the field. As an example, in one delivered plan the leading and trailing leaves had introduced errors of +5.0 mm and −5.0 mm respectively, leading to two data points in Fig. 8. Error bars show the standard deviation of all measurement time points during the delivery of the beam. Note that the deviations were introduced across the entire field; only one leaf pair analysis is presented here for conciseness. The leaf positions were measured using the 50% dose level and linear interpolation between adjacent diodes. The measured values agreed very well with the known errors: when averaged across all data points, detection accuracy was within 0.2 mm on average (Fig. 8) with a maximum deviation of 1.22 mm. Standard deviations ranged from 0.14 mm to 0.34 mm, indicating submillimeter detection precision. Regression analysis showed excellent linearity between introduced and measured errors, with R^2^ >0.998.

## IV. DISCUSSION

A novel gantry‐mounted transmission detector array (TRD) was evaluated for its impact on the radiation dose received by the patient (absorption of radiation, increased surface dose) and its detection sensitivity to a variety of errors (output variation, static MLC positional errors, dynamic MLC positional errors). To begin, is it safe to treat patients with the TRD in the beam path? Approximately 1.1% of a 6 MV beam is absorbed by the array. This is significantly less attenuation than the ion‐chamber‐based TRDs (Compass=3.3%,^(11)^ DAVID = 6.1%–7.2%,^(10)^ and IGM=7% ^(14)^) and similar to the diode‐based (Magic Plate=1% ^(17)^) and optical‐attenuation‐based (1.7%^(16)^) TRDs. The increased surface dose is minimal for fields $\leq 20\times 20\;{\text{cm}}^{2}$ (<4.6%). The collimator scatter increase with field size remains the dominant cause of increasing surface dose with field size. However, caution should be used with any TRD when treating for large fields ($\geq 30\times 30\;{\text{cm}}^{2}$) that already have a significant surface dose. The surface dose increase from this TRD (Delta^4^) is similar to the DAVID and Magic Plate TRDs, and smaller than that reported for the Compass, IQM, and optical TRD devices. Note that the measurement depth for the latter TRDs was shallower (0.01–0.15 mm) than was measured here (0.6 mm) and with David/Magic Plate (0.5–1.5 mm), which may account for the apparent difference. There is no clear relationship between the physical thickness of the TRDs and their transmission/surface dose, as each TRD relies on different materials. It may be worthwhile to include the effect of the TRD in the modeling of the secondary electron distribution in dose calculations,^(12)^ although some clinical implementations use only a tray factor to account for the TRD.^(10)^ Based on our dose profile measurements, the percentage depth dose, in‐plane, and cross‐plane doses were unaffected by the presence of the TRD. Utilization of a tray factor would be sufficient.

Regarding the TRD's performance, the results are encouraging, showing the ability to quantify MLC position deviations as small as 0.7 mm during static or 1.2 mm during dynamic IMRT delivery. An improved interpolation between diodes, as opposed to simple linear interpolation used in this part of the analysis which may introduce additional errors, is expected to further improve the accuracy of leaf‐end detection. The TRD showed very good detection accuracy and precision for leaves at different positions, as well as for different magnitudes of error. In comparison, the DAVID TRD is able to detect leaf position errors of 1.0 mm in static fields and 2.0 mm in IMRT fields.^8^, ^10^ The optical TRD can detect leaf position errors of 1.0 mm in IMRT fields, though paired‐leaf errors were detectable only at the 2.0 mm level.^(16)^ The IQM TRD can detect leaf positions errors of 1.0 mm in static fields (although accuracy seems to decrease for large field sizes, and was only verified up to $15\times 15\;{\text{cm}}^{2}$) and 3.0 mm in IMRT fields.^(14)^ No data are currently available on the leaf position detection accuracy of the Compass system.

The TRD provides information to the QA software every 25 ms. Varian accelerators have a maximum leaf speed of 2.5 cm/s and a maximum dose rate of 600 MU/min (beams with flattening filters). At a sampling rate of 25 ms, the furthest distance a leaf could travel would be 0.625 mm and the greatest MU delivered would be 0.25 MU. There would be improved MLC leaf edge determination accuracy for slower MLC speed/lower dose rates. The transmission detector measurement packets are associated with the appropriate MLC control point based on either tracking the delivered monitor units or a varying gantry angle. There are typically multiple data packets per control point; a representative one is selected by the software (currently the first data packet associated with the control point). To fulfill its potential to act as an independent, real‐time QA device, the software processing speed will need to be significantly increased. Currently field‐specific results are only available to the end‐user after the field has been completely delivered.

The current dimension of the TRD adds ≥5 cm to the gantry towards the isocenter. This reduces the clearance and could limit the usage of the TRD during treatment for some sites. It would be desirable to reduce the detector thickness and locate it within the protected zone of machine's anticollision protection system.

This study used static and dynamic IMRT fields at a fixed gantry angle (0°). Future work will expand this analysis to the variety of plans used in a clinical setting, including the effects of gantry motion (volumetric‐modulated arc therapy) and nonflat beam profiles (flattening filter‐free beams).

## V. CONCLUSION

We have evaluated a new 2D diode‐based transmission detector, with 2.5 mm resolution over the central 25 cm×20 cm area of the treatment field. This detector array has a small effect on the beam transmission/surface dose and successfully detected and quantified machine output deviations and MLC position offsets as low as 1% and 1 mm. The transmission diode array's detection accuracy and precision for MLC positioning errors with both static and dynamic delivery are within 0.7 mm and 1.2 mm, respectively. Overall this 2D transmission detector array is a suitable candidate for online delivery monitoring.

## ACKNOWLEDGMENTS

This work was partially supported by a research grant from ScandiDos AB, Uppsala, Sweden.

## COPYRIGHT

This work is licensed under a Creative Commons Attribution 3.0 Unported License.
